# Editorial: Advancing therapeutics for Alzheimer's disease and related dementias through multi-omics data analysis in ethnically diverse populations

**DOI:** 10.3389/fnmol.2025.1767630

**Published:** 2026-01-12

**Authors:** Anjali Garg, Ravindra Kumar, Rajan Shrivastava, Deepesh Kumar Gupta, Bandana Kumari, Manish Kumar

**Affiliations:** 1 Department of Psychiatry, Washington University School of Medicine, St. Louis, MO, United States; 2 Comparative Oncology Program, Center for Cancer Research, National Cancer Institute, National Institutes of Health, Bethesda, MD, United States; 3 Department of Analytics, School of Computer Science and Engineering, Vellore Institute of Technology, Vellore, India; 4 Division of Oncology, Department of Medicine, Washington University School of Medicine, St. Louis, MO, United States; 5 RNA Biology Laboratory, Center for Cancer Research, National Cancer Institute, National Institutes of Health, Frederick, MD, United States; 6 Department of Biophysics, University of Delhi South Campus, New Delhi, India

**Keywords:** Alzheimer's disease and dementia, biomarkers, machine learning (ML), microglia (MG), multi-omics, therapeutic targets

**Figure 1:**
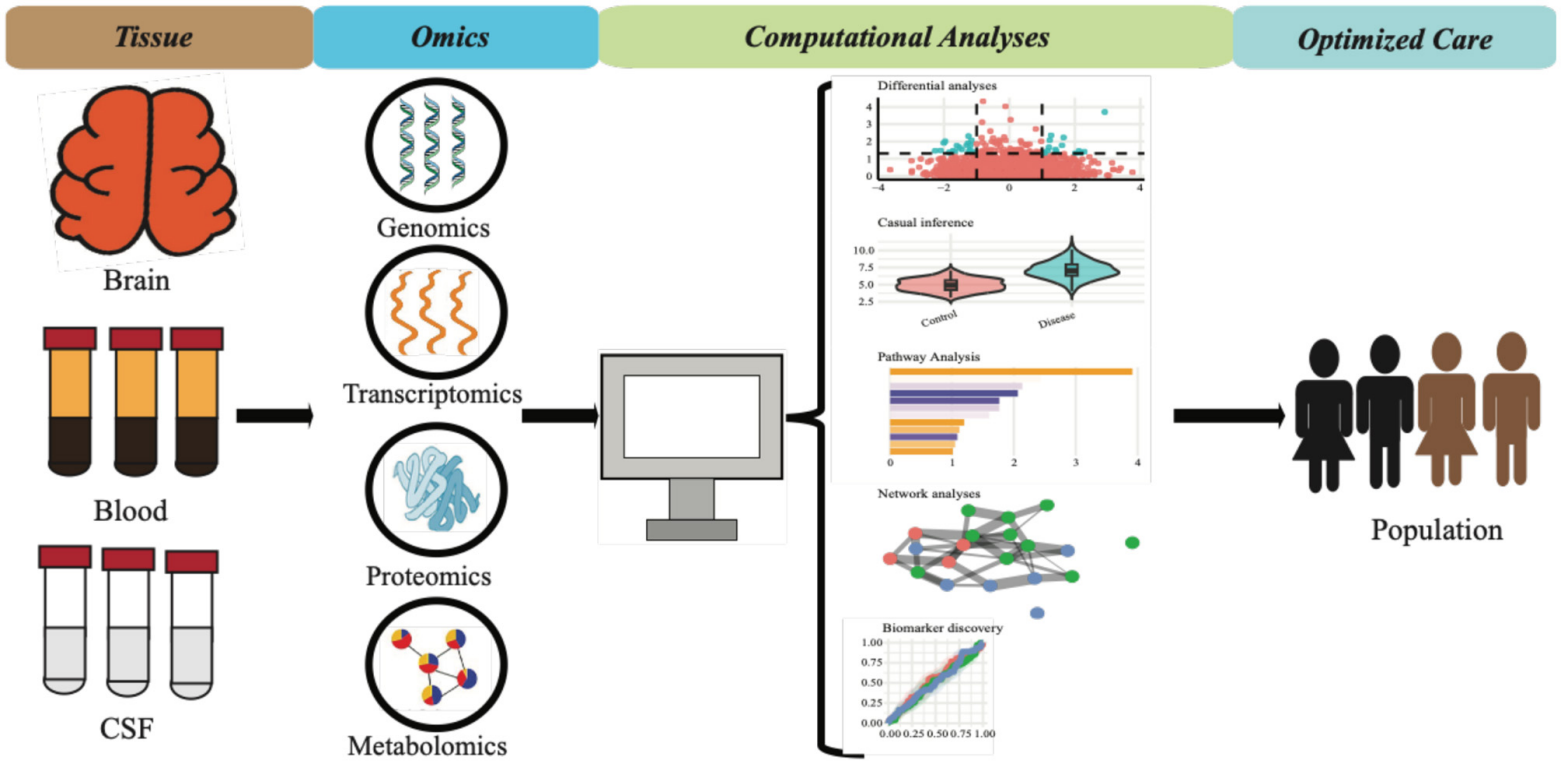
Multi-omics approaches in AD research.

Alzheimer's disease (AD) is a neurodegenerative disorder characterized by progressive cognitive decline and the accumulation of extracellular amyloid-beta (Aβ) plaques and intracellular neurofibrillary tangles in the brain tissue. Like other dementias, AD disproportionately affects certain racial and ethnic groups, remaining a major global health challenge that demands improved strategies for early detection, mechanistic understanding, and therapeutic intervention. This Research Topic focuses on studies investigating microglia dysfunction in various neurological disorders, including AD, while considering factors like experimental models, sex, age, and ethnicity. These studies leverage advanced computational methods to integrate and analyze molecular data. Such approaches, including multi-omics analyses (shown in Figure 1), are elucidating AD's molecular landscape and enabling personalized strategies for neurodegenerative diseases, such as developing genetic and molecular biomarkers, identifying novel therapeutic targets, and designing more effective clinical trials. Ultimately, this research is essential for advancing our understanding of microglial function and dysfunction across diverse populations.

In this Research Topic, we included six articles, including two reviews and four research articles, discussed briefly below. Two studies focus on the marker identification, highlighting potential for improved diagnosis and therapeutic targeting in AD. Wu, Zhang et.al. demonstrate that blood-derived cell-free RNA (cfRNA) can serve as a non-invasive biomarker for detecting molecular changes associated with AD. By integrating cfRNA profiles with single-cell transcriptomic (scRNA) and machine learning (ML) approaches, the authors identified a panel of 34 signature genes, achieving robust diagnostic performance (~90% AUC). These findings underscore the potential of these signature genes for early, non-invasive AD detection and patient stratification. Xu et al. demonstrate how mitochondrial dysfunction serves as a unifying yet divergent element in the pathobiology of AD and glioblastoma (GBM). Single-cell analyses uncovered distinct cell type signatures, with *LPAR1*, regulating mitochondrial dynamics and inflammation in AD, and promoted invasion and altered bioenergetics in GBM. Further machine learning integration identified four key genes namely, *EFHD1, SASH1, FAM110B*, and *SLC25A18* as central mitochondrial regulators. These markers show potential for improved diagnosis and therapeutic targeting although experimental validation and larger cohorts are needed to confirm their clinical relevance. Overall, single-cell deep profiling reveals key regulatory biomarkers and pathways that could improve early diagnosis and therapeutic targeting. Zhang et al. analyzed the relationship between atorvastatin and memory function by integrating data from the National Health and Nutrition Examination Survey (NHANES) and the Food and Drug Administration Adverse Event Reporting System (FAERS). Atorvastatin, a statin drug approved in 2003, is primarily proposed for treating hypercholesterolemia and preventing cardiovascular disease. NHANES findings suggested a potential protective effect of atorvastatin against memory decline while FAERS data revealed specific cognitive adverse events associated with atorvastatin use. Consequently, clinicians and patients should consider atorvastatin's potential benefits against its potential cognitive risks, accounting for individual patient variability, drug responsiveness and lifestyle factors, and implementing appropriate monitoring protocols.

Also, three articles described the early screening and physical therapy-based method to improve quality of life in AD patients. Eliküçük et al. highlighted that bedside and instrumental swallowing assessments are effective for early detection of dysphagia, especially in older adults. In ICU patients, however, dysphagia is often overlooked due to cognitive and behavioral issues. Aspiration pneumonia remains a leading cause of death in this group. The authors noted that early screening and exercise-based swallowing therapy can reduce complications. They also emphasized the importance of diet modification and caregiver education as preventive strategies. Swallowing therapy may shorten hospital stays and improve outcomes while PEG tubes show no clear benefit for survival or quality of life. Malnutrition and poor functional status worsen quality of life, highlighting the need for better-designed studies on dysphagia and nutrition interventions.

Wu, Teng et al. compared the effectiveness of five physiotherapy interventions in the context of AD network meta-analysis. They found that game therapy and acupuncture therapy improved mental wellbeing and the ability to perform daily activities, with acupuncture producing particularly notable gains in cognitive performance. In addition, music therapy and exercise therapy also contribute cognitive benefits and improvement in function outcomes, respectively. Wu, Teng et al.'s analysis demonstrated the significance of personalized physiotherapy and psychotherapeutic approaches in AD care. They also illustrated some of the therapeutic effects that were cognitive, including neuroplasticity and increased blood flow, and the reduction of amyloid protein. Overall, their findings support the use of individualized physiotherapy treatment programs to improve quality of life in AD patients. Adak et al. conducted an extensive review of AD screening models focused on early identification of at-risk individuals before clinical symptoms appear. The review encompasses a broad spectrum of *in vivo* and *in vitro* models, including pharmacologically induced and transgenic animal models, as well as neuronal cell cultures models. These models are essential for studying key AD pathophysiological mechanisms such as tau pathology, Aβ aggregation, synaptic dysfunction, and neuroinflammation. *In vivo* models involve behavioral changes in live rodents, like passive and active avoidance tests and discrimination learning. *In vitro* models use cell lines and primary neuronal cultures to provide controlled environments for investigating cellular mechanisms and drug effects. Overall studies emphasize the value of early clinical screening, personalized physiotherapy approaches in advancing early detection and therapeutic development for AD. The review highlights the strengths and limitations of each model type and discusses how they contribute to the development and assessment of potential AD therapies. This comprehensive review could guide future research on AD screening strategies.

Across these six studies, authors highlight advances ranging from blood-based molecular biomarkers and single-cell transcriptomics to physiotherapy, and screening models, all contributing to better detection and understanding of AD. Together, these findings suggest that integrating multi-omics, clinical assessment, and personalized therapeutic approaches may improve early diagnosis, patient care, and the development of targeted interventions.

